# Total and Subtotal Parathyroidectomy in Young Patients With Multiple Endocrine Neoplasia Type 1-Related Primary Hyperparathyroidism: Potential Post-surgical Benefits and Complications

**DOI:** 10.3389/fendo.2018.00558

**Published:** 2018-09-24

**Authors:** Francesco Tonelli, Francesca Marini, Francesca Giusti, Maria Luisa Brandi

**Affiliations:** Department of Surgery and Translational Medicine, University of Florence, Florence, Italy

**Keywords:** multiple endocrine neoplasia type 1, primary hyperparathyroidism, parathyroid adenoma, parathyroidectomy, young MEN1 patients

## Abstract

**Background:** The choice of surgical treatment for patients with Multiple Endocrine Neoplasia type 1 (MEN1)-related primary hyperparathyroidism (PHPT) remains controversial and it has not been specifically addressed in young patients.

**Methods:** This is a retrospective case series study. The study includes the surgical data and the follow-up of 38 patients younger than 30 years of age, all diagnosed with MEN1, collected and followed-up between 1991 and 2017 at the Regional Referral Center for Inherited Endocrine Tumors of the Tuscany Region, and operated by parathyroidectomy. Genetic and/or clinical MEN1 diagnosis was made before surgery in all patients. Subtotal (9/38 patients) or total parathyroidectomy with auto-transplantation (28/38 patients) were performed in all patients but one, in whom a single mediastinal adenoma was excised from the aorto-pulmonary window. All patients but one, who was operated in 2017, had a post-operatory follow-up of at least 12 months.

**Results:** Total parathyroidectomy (TPTX), with auto-transplantation, was the most frequently adopted operation both as primary (20/38 patients) and secondary (8/38 patients) surgery, followed by subtotal parathyroidectomy (SPTX; 9/38 patients) and limited parathyroidectomy (1/38 patient). At follow-up, lasting a mean of 11.8 ± 6.6 years (range 0–23 years), no persistent PHPT was observed. PHPT recurred in 4/28 TPTX (14%) and in 2/9 SPTX (22%). Permanent hypoparathyroidism showed no statistically significant difference between the procedures (2/9 in SPTX and 5/28 in TPTX).

**Conclusions:** Data from this retrospective study showed the efficacy of TPTX for the treatment of MEN1-PHPT, also in adolescent and young patients, showing, in our series, no risk of PHPT permanence and a longer disease-free period and, subsequently, the possibility to postpone re-intervention with respect to both limited PTX and SPTX. The risk of permanent hypoparathyroidism in TPTX was comparable to STPX, and could be mitigated over the years.

## Introduction

Primary PHPT (hyperparathyroidism) is the most common and, usually, the earliest endocrine manifestation in Multiple Endocrine Neoplasia type 1 (MEN1).

Both timing and type of surgery for MEN1-PHPT remain controversial and rarely investigated by clinical randomized trials. The most common choice is to operate symptomatic cases (1). Parathyroid surgery not only grants the restoration of normal serum parathyroid hormone (PTH) and calcium level, but is also beneficial for the control of gastrin oversecretion in MEN1 patients with a concomitant active gastrinoma (2). Post-surgical restoration of normal calcemia demonstrated also to promote short-term partial recovery of trabecular bone mineral density at lumbar spine and femoral neck and to strongly reduce the manifestation of nephrolithiasis and related secondary kidney co-morbidities (3). Two of the most frequently performed parathyroid surgical interventions in MEN1-PHPT are: subtotal parathyroidectomy (SPTX), consisting in the removal of three/three and a half glands, leaving in site the entire, or a remnant similar-size piece, of the fourth non-enlarged parathyroid gland; or total parathyroidectomy (TPTX) with autologous graft of pieces of the apparently non adenomatous parathyroid tissue in the non-dominant forearm (4). Trans-cervical thymectomy is usually performed, during both these procedures, to prevent thymic carcinoid and remove possible ectopic parathyroid glands (5). However, some reports have shown that limited excision of the affected parathyroid glands could obtain the same result in correcting the hypercalcemia, with a lower risk of persistent hypoparathyroidism (6–8).

The PHPT age of onset is usually during the third decade of life; mean age of surgery is after 35 years of age for most of the patients. However, early onset of PHPT can occur in children and adolescents affected by MEN1 syndrome. Until now, the type and the result of surgery employed in young patients for the treatment of MEN1-PHPT have rarely been addressed in large series of patients, except for a recent study by Goudet et al. which revised a wide population of patients under the age of 21 (9). However, it is not yet clear if young patients have an aggressive type of PHPT responsible for persistence or recurrence of PHPT more frequently than older patients, and if the same MEN1 guidelines applied to adults must be followed also for young individuals (1). In this paper, we describe our surgical experience with MEN1-PHPT patients younger than 30 years, collected, clinically managed, and followed-up over the past 26 years, at the Regional Referral Center for Inherited Endocrine Tumors of the Tuscany Region.

## Patients and methods

### Patients

This study was approved by the Review Board of the “*Area Vasta Centro, Regione Toscana*” at the “*Azienda Ospedaliera-Universitaria Careggi*,” Florence (*Rif. CEAVC OSS 16.234*), and all patients (or legal tutors for individuals < 18 years) gave informed consent for data collection and analysis. Each patient was identified by a study-specific anonymous identification number.

Patients with MEN1-PHPT who underwent a first parathyroidectomy (PTX) before 30 years of age were collected, clinically managed, and followed-up over the past 26 years at the Regional Referral Center for Inherited Endocrine Tumors of the Tuscany Region, and operated on by our surgical team between 1991 and 2017.

This is a retrospective case series. Patients were included in the MEN1 database if they had a genetically proved MEN1, or if they showed at least two of the three main manifestations of MEN1 syndrome (i.e., PHPT, anterior pituitary adenoma, and neuroendocrine tumors of the gastro-entero-pancreatic tract), or developed one of the three main manifestations and had a first-degree relative affected by MEN1 (2).

Sanger's sequencing analysis of the *MEN1* gene was performed in all these subjects. Index cases were analyzed by sequencing of the coding region (exons 2–10) and splicing sites of the gene, while relatives of a mutated *MEN1* patient were screened only for the family-specific *MEN1* mutation. One sequencing-negative patient was further screened by Multiplex Ligation-dependent Probe Amplification (MLPA). Two sequencing-negative brothers were screened by microsatellite-based family linkage at the 11q13 locus.

The majority of these patients were surgically treated by the same surgeon (F.T.). Nine patients, previously operated in other institutions, were submitted to neck re-exploration (secondary parathyroid operation performed within a mean of 6 years from the first surgery) after a previous limited PTX (7/9 patients), which had consisted, in all but one of the cases, in the removal of a single parathyroid gland, or a previous SPTX (2/9 patients). Four were re-operated because of a neck persistent PHPT (persistence of post-operatory elevated serum level of PTH) and five because of a neck recurrent PHPT (recurrence of elevated serum level of PTH months/years after parathyroid surgery).

In all patients, evaluation of the parathyroid glands was performed by echo-color Doppler ultrasonography and cervical and mediastinal scintigraphy by Tc-99m sestamibi. Additional localizing examinations were performed selectively, as clinically indicated, by magnetic resonance imaging (MRI), merged images of technetium-99-m-MIBI SPECT/CT (single photon emission computed tomography/computed tomography), 4D computed tomography (4DCT) and Casanova test (a specific intact PTH-dosing test, able to discriminate between the graft-bearing arm and the neck as the site of the hyperparathyroidism recurrence).

### Surgical interventions

The standard surgical approach was by a cervical incision, performing a bilateral exploration with isolation of inferior laryngeal nerves and an attempt to identify all the parathyroid glands. Two procedures are employed: the SPTX, leaving an entire or a part of one parathyroid gland in the neck (the one appearing not to be enlarged), and marking the site with a metallic clip; or TPTX, removing all the parathyroid glands and grafting fragments of the parathyroid gland appearing as non-adenomatous. From 8 to 10 pieces (about 1 mm^3^ in volume) of fresh parathyroid tissue were transplanted into pockets between the muscular fibers of the brachio-radial muscle of the non-dominant forearm, at the end of neck operation. In some patients, the subcutaneous adipose tissue of the volar forearm was preferred as site of transplant. The time from the tissue collection to the transplant was 58 ± 13 min; parathyroid tissue was preserved in sterile lactated Ringer at +4°C. Efforts were made to recognize or confirm, on the basis of the preoperative localization study, ectopic and/or supernumerary glands, and remove them. The thymus was also removed in all patients by trans cervical approach.

### Intra-operative PTH monitoring

Intra-operative PTH monitoring was accessible to all parathyroid operated patients at the “*Azienda Ospedaliera-Universitaria Careggi*” from 1990. In all our patients, the quick intra-operative determination of intact PTH was determined at the induction of anesthesia, and subsequently just before and at 10–20 min after excision of each gland. The last sampling was performed at the end of surgery, 30–60 min after excision of the last parathyroid gland, in order to have the data before performing the parathyroid implant (10).

### Follow-up

All patients but one, who was operated in 2017, had a post-operatory follow-up of at least 12 months (mean 11.8 ± 6.6 years; range 0–23 years). The serum calcium and PTH concentrations were monitored the first post-operative day and every month after discharge. Persistent PHPT was defined if the following parameters were present during the initial 6 months after the operation: (1) reproducible hypercalcemia and elevated or inappropriate PTH levels; (2) absence of normocalcemic intervals. Recurrent PHPT was defined when calcemia and PTH levels increased 6 months or more after post-operative normocalcemia. Permanent hypoparathyroidism was defined when hypocalcemia (serum ionized or albumin-corrected calcium levels below 1.10 or 2.20 mmol/L, respectively) persisted beyond the first postoperative 6 months, requiring calcium and vitamin D substitution therapy (11).

### Statistical analysis

Clinical and surgical data, age at diagnosis and at surgical intervention, post-operatory outcomes and complications were all analyzed by descriptive statistics; data are presented as nominal categories, percentages, or mean ± standard deviation (SD). Differences in the mean age of MEN1 patients at surgery between the different types of surgery were analyzed by the Student's *t*-test; *p* < 0.05 was considered as the statistical significance. Differences in recurrence rate and permanent hypoparathyroidism rate between TPTX and SPTX were analyzed by Chi-squared test with Yates' correction; *p* < 0.05 was considered as the statistical significance.

## Results

Thirty-eight patients (26 females and 12 males) developed MEN1-associated PHPT before the age of 30 years (mean age of PHPT clinical diagnosis 22.7 ± 5.3 years; range 13–30 years) and underwent PTX at our referral center as primary (29/38 patients) or secondary (9/38 patients) operation.

Genetic screening identified 33/38 *MEN1* mutated individuals by Sanger's sequencing, one patient as carrier of a large intragenic deletion spanning more than one exon of the gene by MLPA and two brothers as carriers of a family-specific MEN1-predisposing haplotype by family linkage analysis of 11q13 locus.

Our series included 11/38 MEN1 single cases (9/11 with an identified *MEN1* gene mutation, and two without an identified germline *MEN1* mutation by Sanger sequencing, but clinically diagnosed with MEN1) and 27/38 familial MEN1 cases from 18 different kindreds (25/27 with an identified *MEN1* gene mutation and 2 without an identified germline *MEN1* mutation by Sanger sequencing but with a MEN1 clinical diagnosis, a family history of MEN1 and a positive 11q13 family linkage analysis). Sixteen patients were referred to our endocrine referral center as affected index cases (11 MEN1 single cases and 5 MEN1 familial case).

The mean age of MEN1 patients at surgery was 23.2 ± 4.9 years (range 13–30 years). No statistically significant difference regarding the age of the patients between the different types of surgery was found. Thirty patients (73.2%) underwent PTX the same year as PHPT clinical diagnosis (0-year gap). The mean gap between PHPT clinical diagnosis and surgery was 0.8 ± 1.9 years.

TPTX was the most commonly employed operation (28/38 patients), followed by SPTX (9/38 patients) and limited PTX (1/38 patient). TPTX was performed as first intervention in 20/28 patients, as a secondary intervention after a previous limited PTX in 6/28 patients, and after a previous SPTX in 2/28 patients. SPTX was performed as a first operation in 8/9 patients and as a secondary intervention 1/9 patient, after a limited PTX, during which two parathyroid glands were removed.

Main characteristics and post-surgical outcomes of SPTX and TPTX are reported in Table 1.

**Table 1:** Clinical characteristics, type of surgery, and post-surgical outcomes of 37 young MEN1 patients who underwent SPTX or TPTX.

	**SPTX (≥3 glands removed)**	**TPTX (all parathyroids removed)**
n. patients	9	28
Previous PTX (n. patients)	1	8
Gender (M/F ratio)	4/5	7/21
Age at surgery (mean ± standard deviation) Range	Primary SPTX: 24.7 ± 3.3 19–30 years Secondary SPTX: 21 years	Primary TPTX: 23.1 ± 5.3 13–30 years Secondary TPTX: 31.7 ± 5.4 24–42 years
PHPT post-operative persistence	0	0
PHPT post-operative recurrence	2 (22.2%)	4 (14.3%)
Time to recurrence (years)	7.0 ± 2.0 5–9 years	10.7 ± 0.8 10–12 years
Site of recurrence (n.)	Neck (2)	Neck (1) Forearm (3)
n. PTX re-interventions	2 (22.2%)	3 (10.7%)^*^
Post-surgical permanent hypoparathyroidism	2 (22.2%)	5 (17.9%)
Years of follow-up after surgery (mean ± SD) Range	First SPTX: 7.8 ± 7.7 0–22 years	13.1 ± 5.7 1–23 years

*PTX, parathyroidectomy; SPTX, subtotal parathyroidectomy; TPTX, total parathyroidectomy; PHPT, primary hyperparathyroidism; SD, standard deviation*.

* *One of four patients presenting recurrent PHPT has normal calcemia and has not undergone parathyroid re-intervention at the time of this study (she was treated with Cinacalcet)*.

The only patient referred for a limited PTX was a 17-year-old boy affected by a single adenoma of a mediastinal ectopic parathyroid gland sited in the aorto-pulmonary window, removed through a left thoracotomy. The patient showed no sign of PHPT persistence or recurrence, and neck parathyroid glands were not affected at the time of this study, 3 years after surgery.

No damage of recurrent laryngeal nerve, PHPT persistence, or complications were observed during the first surgery in all our 37 patients, both for SPTX and TPTX.

At the follow-up, recurrence of PHPT was observed in 2/9 SPTX (22.2%) and in 4/28 TPTX (14.3%). No statistically significant difference in recurrence rate was observed between SPTX and TPTX (*p* = 1.00). The two recurrences after SPTX were both re-operated at the neck, performing a secondary TPTX. Three of four recurrences (75%) after the TPTX occurred at the forearm transplant site. Two of these patients were referred for a second intervention to remove part of the transplant in the forearm; the third was treated with *Cinacalcet*. The fourth recurrence after TPTX occurred in the neck, because of an adenoma in a fifth supernumerary parathyroid gland, which had not been identified and removed during the first TPTX. The second TPTX consisted of a cervical re-operation to remove a neck-located adenoma; this intervention resulted to be particularly difficult, causing a temporary paralysis of the recurrent nerve and rupturing the adjacent carotid artery, which was repaired with a graft.

Post-operatory temporary hypoparathyroidism was observed in all the 28 TPTX patients and in 2/9 SPTX. It regressed spontaneously after 3–4 months in 23/28 (82.1%) of TPTX-operated patients; conversely, 2/9 SPTX (22.2%) and 5/28 TPTX (17.9%) manifested permanent hypoparathyroidism. Permanent hypoparathyroidism rate showed no statistically significant difference between SPTX and TPTX (*p* = 0.84).

No association has been found between type and localization of *MEN1* mutations and PHPT clinical presentation and/or response to surgery, or between patients with a positive or a negative genetic test.

## Discussion

MEN1 endocrine tumors are caused by germline inactivating mutations of the tumor suppressor gene *MEN1* (12). According to the “two hits” mechanism of action for tumor suppressor genes, the second wild type copy of the gene is lost or inactivated at the somatic level in tumors. This process happen independently in the four parathyroids, and it explains why the growth of the parathyroid glands is differently and asynchronously affected. At least one parathyroid gland is, usually, found macroscopically “normal” in 10–20% of the MEN1 patients, at the time of surgical exploration for the treatment of PHPT (13, 14). Furthermore, between 11.9 and 16% of the parathyroid glands removed and histologically examined are normal in MEN1 patients at the time of the PTX (7, 14). Interestingly, no correlation has been found between the volume of the parathyroid glands and the age of the patients with MEN1-related PHPT (13).

The surgical treatment of PHPT in MEN1 patients is usually a compromise between leaving non adenomatous parathyroid tissue sufficient enough to avoid a permanent hypoparathyroidism and removing all the tumor glands to prevent persistence or prompt recurrence of PHPT. This goal can be better achieved either with SPTX or TPTX. In both these procedures, the approximate volume of non tumoral parathyroid gland must be preserved or implanted (11, 15). However, the asynchronous and asymmetrical growth of the parathyroid glands, the presence of at least one normal parathyroid gland in some patients, the fact that any operation leaving parathyroid tissue implies a risk for recurrent disease, and the frequency of ectopic and/or supernumerary gland higher than expected and often located in non-conventional sites or embedded within the cervical fatty tissue make surgical treatment of MEN1-PHPT extremely challenging.

Also parathyroid fragments, re-implanted after a TPTX, cause recurrence; our experience showed them as the most common site of PHPT recurrence in TPTX. The modalities of transplant are empirical: the number of parathyroid fragments to be implanted is a controversial point. Most surgeons insert at least 20 fragments (16–18), but it is not clear if they are completely vital at the moment of the implant. Our approach utilizes fresh parathyroid tissue, rigorously maintained at +4°C between explant and re-implantation, to reduce to a minimum the ischemic time. According to our experience, 10 fragments of about 1 mm^3^ in volume, are enough to guarantee that they take root and maintain an adequate parathyroid function. However, research on the ideal number of fragments and the modality of implant should be implemented to clarify this technical point.

The revision of several surgical experiences on wide series of patients, published in recent years, showed clearly that persistent PHPT is more frequent after SPTX than after TPTX; meanwhile, recurrent PHPT has a similar frequency for the two types of surgery (19). However, the rate of PHPT recurrence is strongly dependent on the length of follow-up, as recurrences happen earlier after SPTX than TPTX (15, 19, 20). These data are confirmed by a meta-analysis (21) and by the only randomized clinical trial performed until now (18). All these studies also highlighted how both transitory and permanent hypoparathyroidism is more frequent after TPTX than SPTX. However, hypoparathyroidism may improve progressively with time, as demonstrated in patients with the longest follow-up in absence of a replacement therapy (15).

Very little information about surgical treatment of PHPT in young patients affected by MEN1 syndrome is available in literature; surgical experience of MEN1-PHPT has been globally referred to all the patients. The *Group d'étude des tumeurs endocrines* has recently revised a large population of patients aging <21 years (9). Out of 160 MEN1 patients, PHPT was diagnosed in 122 (75%). Only 37 of them (30.3%) were operated, either for clinical symptoms or hypercalcemia. Operation before 10 years of age was very rare in this experience (only one patient). Half of these patients were operated with SPTX, 26% with single parathyroid gland excision, and 21% with excision of 2 or 2.5 parathyroid glands. At follow-up (the patients were followed until 21 years of age), which was completed for only 67%, most of the patients were normocalcemic and only 4 (11%) were hypocalcemic. Persistent or recurrent PHPT was observed in 22% and reoperation was performed in 5%.

It is difficult to establish only from these data if young patients are more prone to persistence or recurrence of PHPT than older patients. In particular, a comparison between SPTX and TPTX was not specifically performed in young patients. The results from our study and from our surgical experience show that TPTX is effective and indicated also in young patients, and it is better to perform an aggressive surgery than a limited PTX. MEN1 patients are at risk of having an inadequate type of surgery, especially when familial PHPT is not suspected and genetic testing has not been performed before surgery. In our surgical experience, we have had the advantage of operating most of the patients already knowing that they were carriers of a form of PHPT due to *MEN1* gene mutation. In all but one patient, TPTX or SPTX were performed. Nine of these interventions were requested as second intervention, after a period of between 3 and 14 years, for 5 PHPT persistence and 4 relapses, secondary to a previous limited PTX or a SPTX, not performed in our center. On the basis of these findings, the hypothesis that at time of the diagnosis of PHPT in young MEN1 patients only one or two parathyroid glands are affected and there is a long time period before the other parathyroid glands become hyperfunctioning is not persuasive. Recently, the NIH experience regarding different surgical approaches to treat PHPT in MEN1 syndrome was referred by Nilubol et al. (22). Patients were studied and followed-up using a strict protocol. Sixteen patients were submitted to a limited PTX (<2 glands): 11 of them had a persistence of PHPT and 3 a recurrence of PHPT. The authors concluded that a limited PTX, even if guided by preoperative localizing studies, is associated with a significantly higher rate of persistent PHPT compared to excision of over 3.5 parathyroid glands. The main cause of the failure of a limited PTX is the inadequacy of the preoperative localizing studies, missing enlarged controlateral parathyroid glands in 86% of the patients. However, other authors have obtained positive results with limited interventions, such as the unilateral clearance (excision of the parathyroid glands and thymus from only one cervical side) (8, 23) or selective parathyroidectomy (7, 24, 25). Most of the patients operated by limited PTX are older, and there is too little follow-up time for the results achieved to be reliable (22).

The reason for early onset of PHPT and greater recurrence after surgery in some patients has not yet been clarified. At this time, it remains controversial whether there is a genotype-phenotype correlation for the severity of PHPT. The Dutch MEN1 study found a lower risk of persistent or recurrent PHPT after less than SPTX in presence of *nonsense* or *frameshift* mutations in exons 2, 9, and 10 (26). Mutation in exon 3 of the *MEN1* gene has been found to be linked to risk of recurrence (15). Our study did not confirm these findings.

From our experience, it has been confirmed that re-intervention at the cervical level can be at risk for serious complications, and it must therefore be avoided as much as possible, performing the most efficacious type of surgery as first operation.

In conclusion, our data showed the efficacy of TPTX for the treatment of MEN1-PHPT, also in adolescent and young patients, showing, in our series, no risk of PHPT permanence and a longer disease-free period and, subsequently, the possibility to postpone re-intervention with respect to both limited PTX and SPTX. The risk of permanent hypoparathyroidism in TPTX was comparable to STPX, and could be mitigated over the years.

## Author contributions

FT wrote the manuscript. FM analyzed patients' data, designed the table, and helped in writing the manuscript. FG collected patients' data, created the patient database, and helped in writing the manuscript. MB designed the study and revised the final version of the manuscript.

### Conflict of interest statement

The authors declare that the research was conducted in the absence of any commercial or financial relationships that could be construed as a potential conflict of interest.
